# Feasibility study of emotion mimicry analysis in human–machine interaction

**DOI:** 10.1038/s41598-025-87688-z

**Published:** 2025-01-31

**Authors:** Herag Arabian, Tamer Abdulbaki Alshirbaji, Ashish Bhave, Verena Wagner-Hartl, Marcel Igel, J. Geoffrey Chase, Knut Moeller

**Affiliations:** 1 Institute of Technical Medicine (ITeM), Furtwangen University, 78054 Villingen-Schwenningen, Germany; 2 Innovation Center Computer Assisted Surgery (ICCAS), University of Leipzig, 04103 Leipzig, Germany; 3 Department of Microsystems Engineering (IMTEK), Faculty of Engineering, University of Freiburg, Georges-Koehler-Allee 101, 79110 Freiburg, Germany; 4 Department of Industrial Technologies, Campus Tuttlingen Furtwangen University, 78532 Tuttlingen, Germany; 5 Imsimity GmbH, 78112 St. Georgen im Schwarzwald, Germany; 6 Department of Mechanical Engineering, University of Canterbury, Christchurch, 8041 New Zealand

**Keywords:** Human behaviour, Patient education, Quality of life, Therapeutics, Software

## Abstract

Health apps have increased in popularity as people increasingly follow the advice these apps provide to enhance physical and mental well-being. One key aspect of improving neurosensory health is identifying and expressing emotions. Emotional intelligence is crucial for maintaining and enhancing social interactions. In this context, a preliminary closed-loop feedback system has been developed to help people project specific emotions by altering their facial expressions. This system is part of a research intervention aimed at therapeutic applications for individuals with autism spectrum disorder. The proposed system functions as a digital mirror, initially displaying an animated avatar’s face expressing a predefined emotion. Users are then asked to mimic the avatar’s expression. During this process, a custom emotion recognition model analyzes the user’s facial expressions and provides feedback on the accuracy of their projection. A small experimental study involving 8 participants tested the system for feasibility, with avatars projecting the six basic emotions and a neutral expression. The study results indicated a positive correlation between the projected facial expressions and the emotions identified by participants. Participants effectively recognized the emotions, with 85.40% accuracy demonstrating the system’s potential in enhancing the well-being of individuals. The participants were also able to mimic the given expression effectively with an accuracy of 46.67%. However, a deficiency in the performance of one of the expressions, surprise, was noticed. In the post processing, this issue was addressed and model enhancements were tailored to boost the performance by ~ 30%. This approach shows promise for therapeutic use and emotional skill development. A further wider experimental study is still required to validate the findings of this study and analyze the impact of modifications made.

## Introduction

In recent years, digital health applications have rapidly expanded, offering significant contributions to physical and mental wellness while encouraging a healthier lifestyle^1–4^. As healthcare systems face increasing strain^5–8^, these applications, along with digital therapies, present a promising solution for both patients and healthcare providers by facilitating digital patient-led care^9,10^. These apps enable a more personalized, patient-centric approach by collecting vital information over extended periods, which can lead to more accurate diagnoses and treatments than the brief interactions typically experienced during medical appointments. Intelligent learning systems also offer the potential to further improve the quality, efficiency, and accessibility of patient care^11^.

To support therapeutic interventions for individuals with autism spectrum disorder (ASD) novel digital emotion recognition tools are required. ASD is a neurodevelopmental disorder that affects an estimated 1–2% of the population, impacting social skills, communication, behavior, and interests^12–15^. It can lead to significant health issues, such as depression and anxiety, due to social isolation and underemployment^12^. Individuals with ASD often face significant challenges in recognizing and interpreting emotional expressions, both in themselves and others. People with ASD may struggle to identify facial expressions, tone of voice, and body language, which are crucial for understanding emotions in social contexts. As a result, they might have trouble responding appropriately to emotional signals, affecting their ability to engage in empathetic or reciprocal social exchanges. These challenges can contribute to social isolation, anxiety, and difficulties in forming relationships^16^. To address these challenges, tailored therapies for various ASD levels are designed, such as single or group sessions.

This study aims to develop and validate a real-time facial emotion recognition model using a geometric-based approach, focusing on facial landmarks for effective emotion classification in therapeutic applications, particularly for individuals with ASD. The new tool being developed involves a closed-loop feedback system, which will immerse users in gamified scenarios that are designed to elicit emotional responses and help them practice social interactions in a virtual environment. By leveraging real-time feedback, the system not only fosters emotional recognition and mimicry but also provides clinicians with essential data to monitor progress and tailor therapy to individual needs. Such digital interventions hold promise for helping individuals with ASD better navigate and master social situations^17–19^.

The definition of emotion is often widely contested^20^. For this research, emotion is viewed as the component process model, where emotions are sequences of events triggered by internal or external stimuli^20^. The understanding of emotional stimuli, particularly their role and influence in clinical contexts, has evolved over time. Emotional stimuli identified as threats significantly impact attention, processing priorities, and Pavlovian responses^21,22^. Recent studies suggest reactions to these stimuli are context-dependent and aligned with personal goals, illustrating the complexity and difficulty in understanding emotional responses^23,24^.

Emotional intelligence (EI) has gained recognition as both a theoretical construct and a critical factor in human functioning. It refers to the ability to recognize, understand, and manage emotions in oneself and others, as well as using emotions to guide thinking, behavior, and decision-making. The concept of EI has been explored in various frameworks, with some models focusing on mental abilities and others emphasizing personality traits. Research has shown that EI is a significant predictor of success in various domains, including personal well-being, mental health, and social functioning. Studies have demonstrated that individuals with higher EI tend to exhibit better emotion regulation, higher social engagement, and enhanced coping mechanisms in the face of stress^25^. While the emotional intelligence literature is extensive, there remains a need for deeper understanding of how specific emotional regulation strategies contribute to overall emotional and social well-being, particularly in real-world settings^26,27^. In the study of emotion and attention, tools like the International Affective Picture System (IAPS) provide standardized emotional stimuli, allowing researchers to explore how emotional cues influence cognitive processes, thus contributing to our understanding of emotional responses in various contexts^28^.

Identifying emotions is challenging due to the diverse ways individuals express them^29^. However, emotions are often communicated through facial expressions (55%), speech and voice patterns (35%), and physiological signals (10%)^30^. Previous research^31,32^ has also shed light on the importance of whole-body expressions in understanding emotions, especially when facial expressions are not visible. The growing interest in non-verbal emotional cues underscores the importance of emotion recognition in enhancing human-machine interactions^33–38^. Facial expressions are often analyzed using two main methods: image-based and geometric-based approaches. This study employs a geometric-based approach, extracting facial landmarks and implementing a simple pattern recognition model to classify emotional states. Using facial landmarks offers a comprehensive approach to emotion recognition. Prior research demonstrated the effectiveness of incorporating facial landmark locations into classification processes, achieving promising results using a unique loss function with a diverse range of emotion datasets^29^. In another study, facial landmarks from a Kinect 3D device were used to identify action units (AUs), achieving 96% accuracy on the Karolinska directed emotional faces (KDEF) dataset^39^. Combining facial landmarks and physiological signals yielded a high 86.94% accuracy in classifying emotions^40^. Selecting key facial landmarks for geometric analysis of facial gestures also yielded strong performance on multiple datasets, with machine learning models achieving up to 97% accuracy on the extended Cohn-Kanade (CK+) dataset using a k-nearest neighbor (KNN) classifier with real-time processing^41^.

A facial expression mimicry experiment is valuable as it enhances the understanding of emotional cues and fosters empathy^42^. Emotional mimicry is not just about replicating muscle movements, but rather interpreting and responding to emotions, which facilitates social bonding and improves interactions. Research in^43^ emphasizes that mimicry is more likely when there is an intention to affiliate, highlighting its importance in establishing social connections. Additionally, Majumder et al.^44^ explores a novel approach for generating empathetic responses in dialogue systems by mimicking user emotions. The study uses a transformer encoder to classify emotions and generate responses that balance empathy and appropriateness, focusing on positive and negative emotional clusters. An experiment was carried out in^39^, where the aim was to recognize the six basic emotional states of joy, sadness, surprise, anger, fear, and disgust, and an additional neutral expression based on facial expressions using a three-dimensional face model. The study used a Microsoft Kinect to create 3D models of participants’ faces and calculated features based on six AUs derived from the facial action coding system (FACS)^45^. Participants in this study were instructed to mimic emotional expressions displayed on a screen while the Kinect recorded their facial movements. The features extracted were classified using a k-nearest neighbors (k-NN) and multilayer perceptron (MLP) neural network classifiers. The experiment involved six male subjects, and each participated in two sessions, resulting in a total of 252 facial expression recordings for analysis^39^. These studies demonstrate the importance of emotional mimicry in both social and technological contexts. While they highlight the benefits of emotional recognition and mimicry, they do not fully address the challenges faced by individuals with conditions like ASD, where emotion recognition and expression can be particularly difficult.

To develop an efficient emotional feedback system, a robust model for expression recognition is essential. This study builds on these insights by developing a system that uses facial expression mimicry to improve emotional recognition, aiming to create more effective therapeutic interventions and emotion-aware AI systems. The proposed model is based on a pattern recognition framework and employs a geometric-based approach, utilizing facial landmarks extracted in real time for emotion classification. Unlike previous approaches that primarily focus on image-based or action unit-based methods, this approach is computationally simpler, allowing for faster processing and making it suitable for real-time applications. Additionally, the use of facial landmarks differentiates this model from other systems that rely on more complex 3D modeling or multimodal data.

To achieve these objectives, a facial emotion recognition model was developed, trained, and validated on a combination of the OULU-CASIA^46^ and FACES^47^ emotion databases. The integration of these two distinct databases introduces variation in external influences such as lighting conditions, camera angles, and subject demographics (e.g., age, gender, ethnicity), which provides a more robust feature space analysis for emotion classification.

In this study, an experiment was designed to evaluate facial expression recognition in real time. The developed model was used in a preliminary, proof-of-concept feasibility trial involving neurotypical participants. During the trial, participants engaged in emotion recognition and mimicry tasks, attempting to replicate avatar-displayed emotions under controlled conditions. The system’s performance was assessed based on classification accuracy and participant feedback, with a focus on identifying challenges and areas for refinement. Full experimental details, including data collection and analysis, are outlined in the “Methods” section.

The experimental results demonstrate that the model is computationally efficient and aligns well with existing theories on emotion recognition. This supports the feasibility of the model for practical, real-world use and provides a simpler alternative to more complex 3D and multimodal methods. The proof-of-concept trial helped identify key issues in complete system development and highlighted areas for improvement. It also collected data for further refinement of the recognition model. Overall, this work demonstrates the feasibility of such a system in real-time settings, laying the groundwork for further development and optimization in future studies.

The paper is divided into the following sections, the “Results” section provides the results from the recognition model, the experiment and post analysis of the recognition model. The “Discussions” section follows with the findings from the respective results. The “Methods” section then follows with the description of the methods used for the model building, feature extraction, experimental setup and analysis criteria.

## Results

### Emotion recognition model

**Fig. 1:**
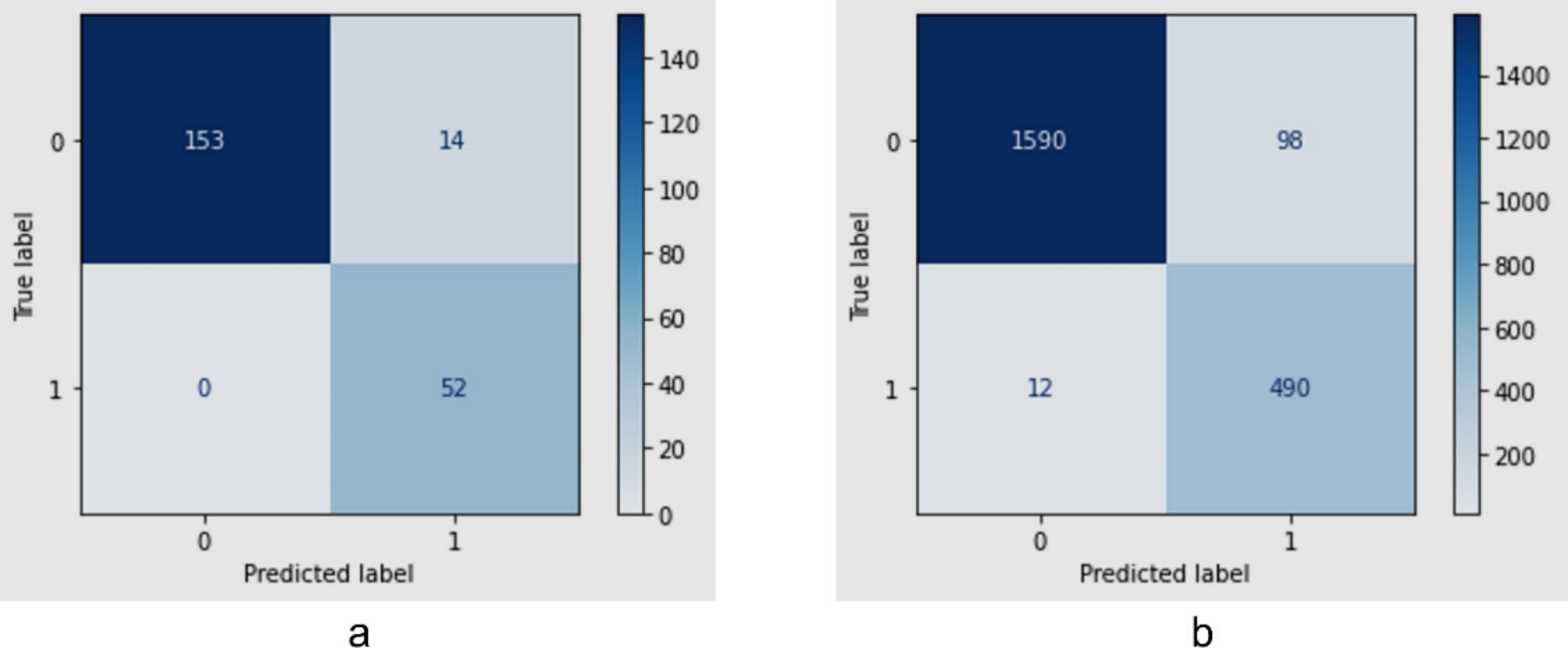
Confusion matrices of the validation set (left **a**), and the entire dataset (right **b**).

The emotion recognition model was trained to provide a two-class output of positive and negative as described in the “Methods” section. The validation set consisted of 219 images with the training set having 1971 images. The model yielded a true positive (TP) accuracy of 93.61%, precision of 94.96%, recall of 93.61% and an F1-score of 93.84% on the validation set. When compared to the entire dataset, i.e., training and validation set combined, the model achieved a TP accuracy of 94.98%, precision of 95.60%, recall of 94.98% and F1-score of 95.11%. In Fig. 1 the confusion matrices are depicted for both the validation set (left a) and the entire dataset (right b).

### Emotion assessment experiment

**Fig. 2:**
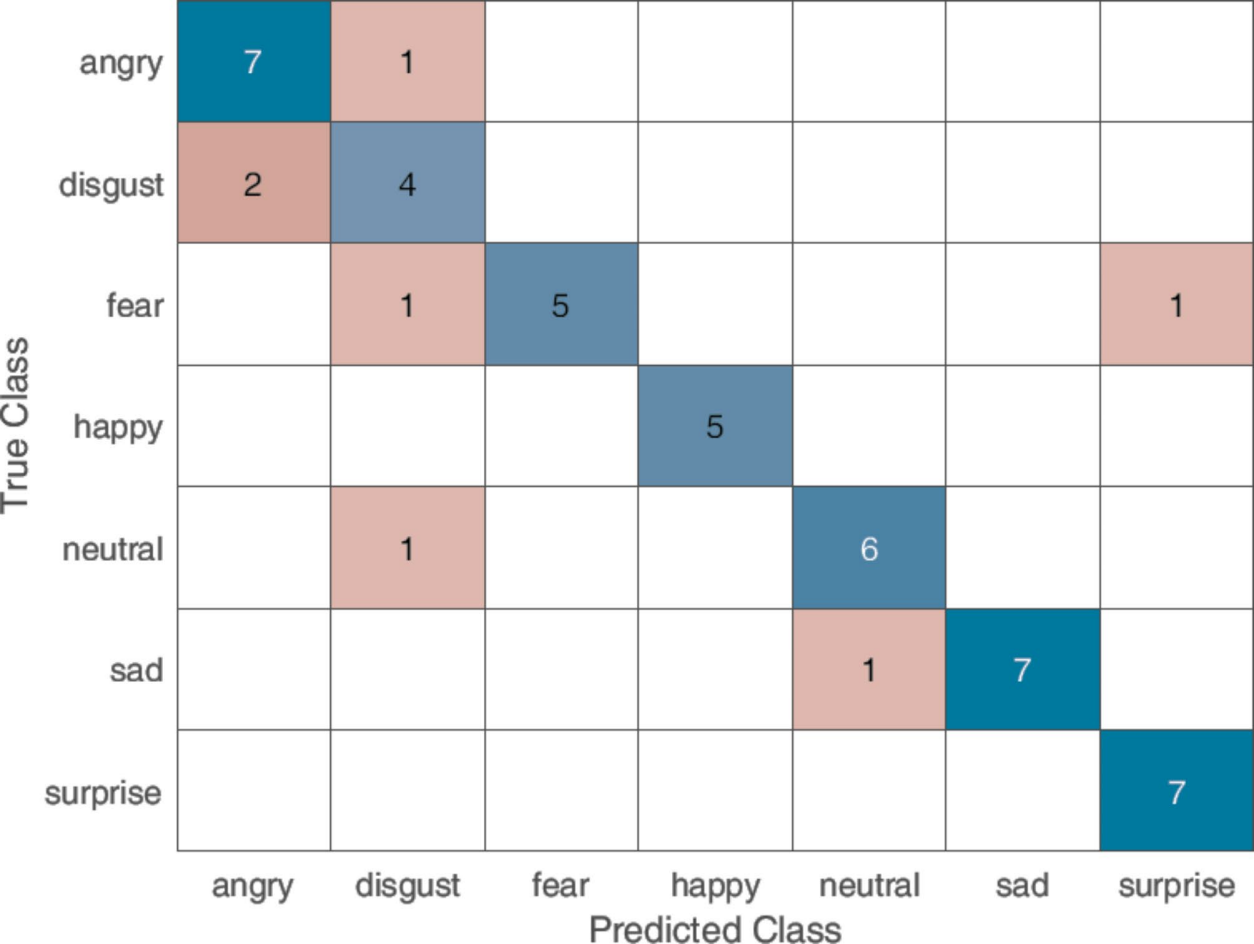
Confusion matrix of the pre-defined facial expression and the participants response. Blue indicates a positive identification; pinkish orange represents a misclassification with intensity of the hue varying with respect to the number of identifications.

The results from the emotional assessment showed a positive identification of the facial expressions with a TP accuracy of 85.40%. In Fig. 2 the confusion matrix between the presented avatar expressions and the participants responses can be seen. The number of observations varies as the participants were shown random sequences of the expressions minus one (i.e., six out of the seven expressions).

### Emotion mimicry experiment

In the emotion mimicry experiment, a first look was performed into the number of times the participant was asked to try to mimic again due to failure to reach the threshold. The ratio of class repetitions with respect to the max number of attempts showed a median of 37.50%, with an inter quartile range (IQR) of 20.83% from 26.04% (25th percentile) to 46.87% (75th percentile). Although the recognition model is set to classify positive and negative, the evaluation was performed on the six + neutral emotion class basis. The surprise class revealed the highest number of repeated attempts at 66.67% with the sad class having the least at 0%. Table 1 shows the distribution and number of times an expression was given the feedback “Try Again!”.

**Table 1:** Results of “try again” feedback given to the participants.

Class name	“Try again” count	% Max attempts
Anger	9	37.50
Disgust	9	37.50
Fear	6	25.00
Happiness	7	29.17
Neutral	12	50.00
Sadness	0	0.00
Surprise	16	66.67

**Fig. 3:**
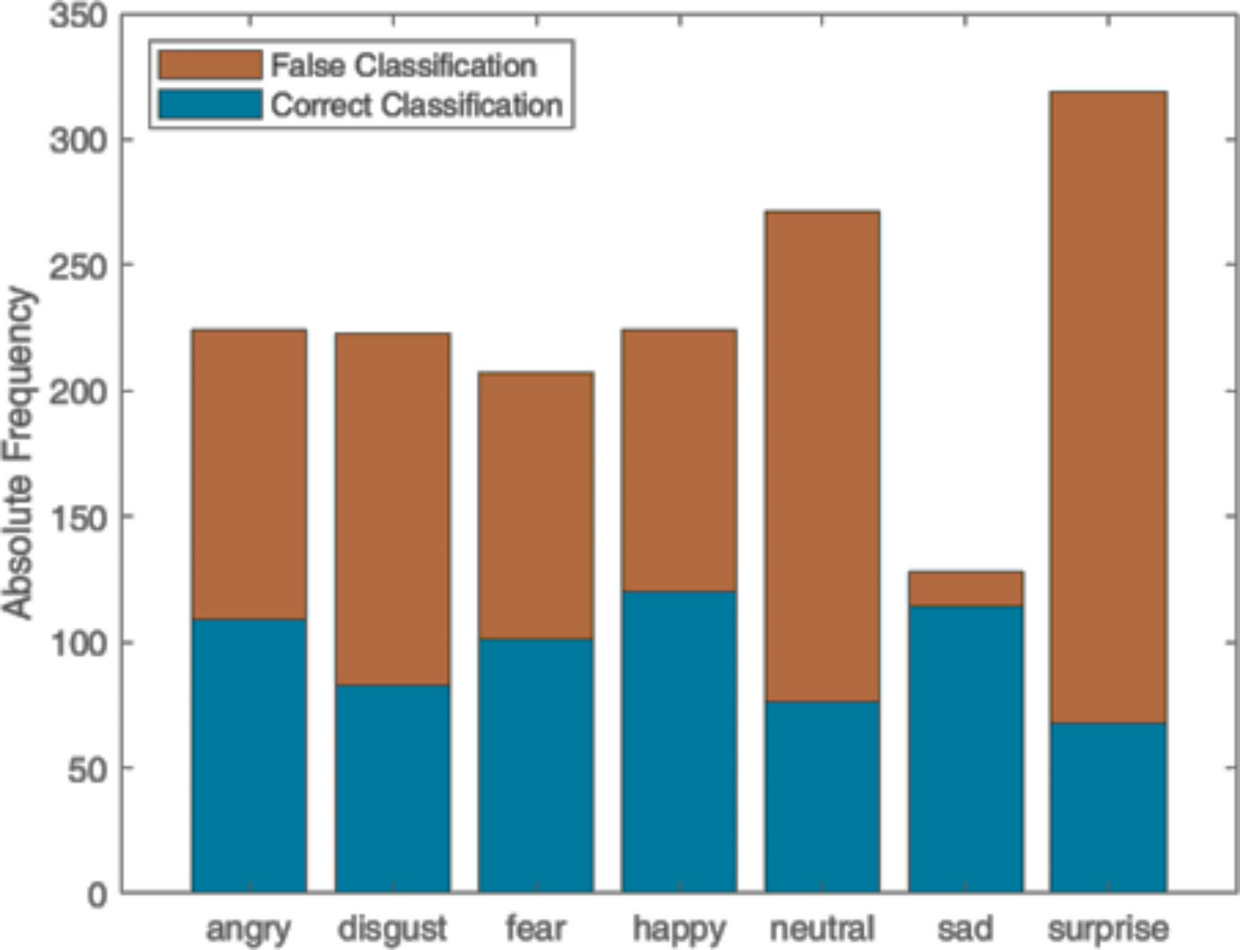
Prediction results from the two-class emotion recognition model visualized for each avatar facial expression. The bars in red indicate a false classification while that in blue represent correct classifications.

Figure 3 shows the classification results of the two-class system for the different avatar facial expressions. As seen in Fig. 3, the surprise class had the highest number in absolute frequency, due to the number of repeat attempts after false classification. The sad class the lowest. Thus, the sad class showed the best ratio of correct classification versus false classification, while the surprise class showed the worst. The large number of instances is related to the frequency in prediction over the period of 5 s, where, in this experiment, the recognition model was operating at an average rate of around 3.2 Hz. The results also show the model achieved a TP accuracy of 46.67% during the experiment.

### Feature post experiment result

**Fig. 4:**
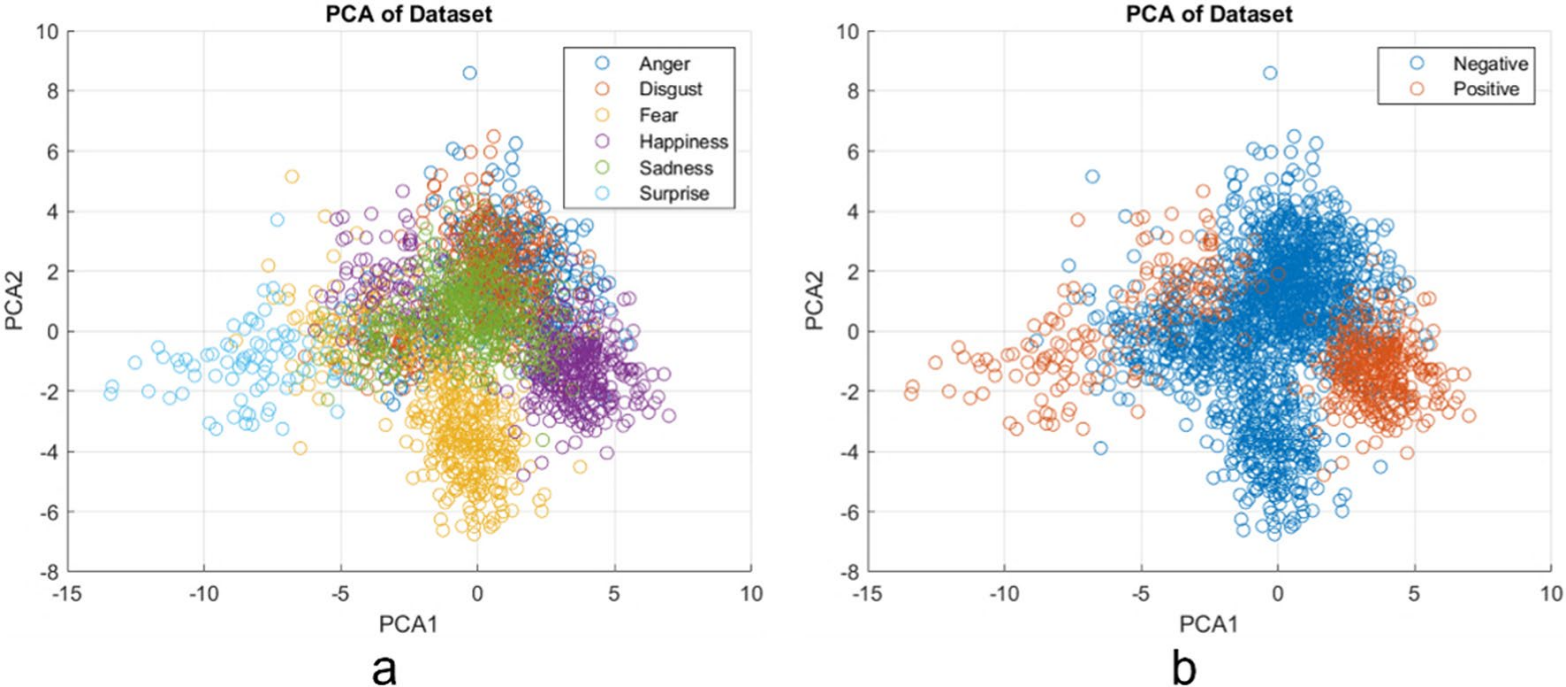
Principal component analysis results from post experiment feature alterations, showing the first two principal components for both the six-class (left **a**), and two-class system (right **b**).

After analyzing the results, a post analysis on the feature space was conducted. An additional distance feature (between landmark points 18 and 106) was added to improve the performance of the recognition model, which was re-trained. In Fig. 4 the results show the features, after performing a principal component analysis (PCA), of two of the principal components for the six classes (left a), while on the right (b) are the same principal components for the two-class system of positive and negative using the new 16 feature space.

## Discussion

The primary goal of this study was to develop and validate a real-time facial emotion recognition model using a geometric-based approach, focusing on facial landmarks for effective emotion classification in therapeutic applications. By leveraging this model, the study aims to provide a simpler, more computationally efficient alternative to complex 3D and multimodal methods, facilitating real-time emotional feedback for individuals with ASD and supporting tailored therapeutic interventions.

The feature reduction process adopted for the emotion recognition model proved to be effective in retaining the essential geometric features necessary for accurate emotion classification. By focusing on the rate of change in distances and angles derived from facial landmarks, a simplified model was obtained without having to compromise accuracy. As illustrated in Fig. 1, the model successfully classified emotion data from the combined OULU-CASIA and FACES datasets. The integration of these two diverse datasets ensured a robust representation and enhanced the model’s capability to capture a wide range of emotional patterns. This approach not only validates the model’s performance across different datasets, but also demonstrates its potential applicability in varied real-world scenarios. Successful implementation of feature reduction underscores the importance of selecting pertinent features which contribute significantly to model accuracy, while maintaining computational efficiency.

Results from the emotion assessment experiment illustrated in Fig. 2 underscore participants’ ability to relate to the virtual characters (avatars) and accurately distinguish between different virtual emotional expressions. However, an interesting pattern emerged in which participants exhibited confusion when navigating various negative emotions (anger, disgust, fear, and sadness). This difficulty is evident in the confusion matrix in Fig. 2, where most errors occurred within the negative classes, such as mistaking anger for disgust or fear for disgust, and even occasionally identifying sadness as neutral. Similarly, the recognition model struggled to differentiate some of these classes from the datasets.

The observed accuracy of 46.67% in mimicking expressions, achieved by the participants, provides an initial reference point for evaluating performance, but its significance must be interpreted within the broader context of existing research on emotional expression and recognition. Prior studies^48^ have highlighted that individuals with ASD often exhibit atypical emotional expressions, which are challenging for both neurotypical (NT) and ASD individuals to recognize. This raises questions about how NT participants’ performance in such tasks compares to that of individuals with ASD, particularly given the differences in emotional representation and expression across these groups. These findings emphasize the need for further studies to compare NT and ASD performance in similar tasks, establishing clearer benchmarks and evaluating how well systems, such as the one proposed, can address these specific challenges.

Examining the feature space map of the principal components in Fig. 4a reveals a clear boundary between the negative emotions of anger, fear, disgust, and sadness is notably challenging. This difficulty is mirrored in the participants’ responses during the emotion assessment, highlighting a broader issue in accurately identifying these negative expressions. Consequently, these findings suggest both human perception and machine learning models face significant hurdles in distinguishing between similar negative emotions, thus requiring more input such as voice or whole-body position^31,32^.

Understanding the social context and intention behind mimicry helps in deciphering the subtle ways in which people connect emotionally. The significance of this work lies in its potential to enhance human-computer interactions by making them more emotionally intelligent and contextually aware. In the six-class avatar expression identification assessment trial, where the emotion to be identified is randomized for each user, collecting data for only five out of the six classes (i.e., number of classes minus one) is a strategic approach to ensure balanced, unbiased, and high-quality data collection. By rotating and ensuring each user identifies only five out of the six expressions, potential biases can be controlled to ensure more balance in the collected data. This approach can also mitigate order effects, such as fatigue or learning biases, which may distort the data, as participant performance varies with respect to time. Thus, by limiting the number of classes each participant interacts with, the risk of these order effects influencing the results is minimized. This approach thus also reduces the cognitive load on participants and can lead to higher quality data as users are less likely to experience fatigue.

The emotion mimicry experiment exposed significant weaknesses in the model’s ability to classify new data from participants. As depicted in Fig. 3, there was a higher rate of misclassifications compared to correct classifications across the various facial expressions. This deficiency is largely attributed to the high rate of emotion expression repetitions, indicating the model’s struggle with robust recognition. While certain expressions, particularly negative ones, are inherently more challenging for participants to replicate accurately, the primary cause for repetition was the model’s limited ability to accurately identify these different classes.

Furthermore, the classification results in Fig. 3 highlight distinct performance patterns of the model. The surprise class exhibited the highest frequency of occurrences and subsequently the worst ratio of correct to false classifications, underscoring the complexity of accurately recognizing this emotion from the given features. In contrast, the sadness class showed the best performance, with the highest ratio of correct classifications. Despite this set of results, the overall moderate accuracy of the model indicates significant room for improvement, especially in recognizing more complex or subtle expressions. These findings emphasize the need to refine the emotion recognition algorithms and adjust threshold settings to enhance overall performance, reducing the necessity for repeated mimicry attempts. Such improvements are important for achieving a more seamless and intuitive user experience.

The addition of the distance feature between landmark points 18 and 106 allowed for more distinct classification of the surprise emotion, as evidenced by the improved separation observed in the principal component analysis (PCA) plot (Fig. 4), which is often subtle and can be easily confused with other emotions. The inclusion of this feature resulted in a clearer differentiation of surprise from other emotional states, which was not as evident in the initial model. Additionally, the significant improvement in accuracy on the collected experimental data (~ 30%) demonstrates the model’s increased sensitivity to the real-time facial expressions, especially when the input feature space is enhanced. The model’s performance increase highlights the importance of refining feature selection to improve real-time emotion recognition, confirming that the adjustments made have positively impacted the system’s robustness and reliability.

### Limitations

With any experiment and study there are limitations. A notable limitation of this study is the relatively low frequency of recognition, which may have impacted the real-time performance of the system. This constraint potentially hindered the ability to accurately capture and classify rapid changes in participants’ facial expressions. Communication difficulties arose from the use of different programming languages for various components of the system, leading to integration challenges and increased latency. These technical issues not only affected seamless operation of the experiment, but also may have introduced errors and inconsistencies in the data collection and analysis processes. Another limitation of this study is the relatively small sample size, which may have an impact on the generalizability of the findings but was effective in demonstrating the potential of the approach without significant burden on subjects and volunteer numbers. However, the limited sample size can signify that the variability and diversity in facial expressions across different demographics and individual differences may not have been fully captured. This study was conducted with neurotypical participants to establish a baseline performance for the model. This approach provided a controlled setting to validate the system’s stability and effectiveness before introducing additional variables. However, this limits the immediate applicability of the findings to individuals with autism, whose unique emotional expressions and recognition patterns may require specific adjustments.

As the primary aim of this study is to establish the fundamental viability of the system for real-time emotion recognition, particularly focusing on the accuracy of classifications under controlled conditions, only the strongest representations were used. This approach provides a first assessment of model performance before adding added complexity or subtlety. However, identifying weaker, more ambiguous emotional expressions is crucial for real-world applications, particularly to benefit individuals with ASD who may face challenges with subtle emotional cues. Thus, incorporating weaker representations is a necessary next step given the results from this pilot study.

Addressing these limitations in future studies will be crucial for enhancing the model’s performance and ensuring more reliable and efficient emotion recognition.

### Future work

The developed system has significant practical implications for various applications, including human-computer interaction, gaming, and mental health monitoring. Its real-time emotion recognition capability can greatly enhance user experience by providing valuable insights into user behavior. Future work will focus on expanding the dataset to include a broader range of emotional states and improving the interface to boost user engagement. Exploring advanced machine learning models and incorporating more features could further enhance the system’s accuracy and robustness.

Future research will also aim to improve the discriminatory power of recognition systems and refine methods to help participants accurately identify subtle differences in negative emotional expressions. This could involve refining training protocols, incorporating additional contextual cues, and developing more sophisticated algorithms for emotion recognition. The model will be further tested and refined with participants on the autism spectrum to ensure it effectively meets their unique needs and addresses specific challenges in emotion recognition. Such advancements highlight the potential for applying these techniques to other emotion recognition tasks, thereby promoting scalability and adaptability in future research.

While this study focuses on the development and evaluation of a real-time facial emotion recognition system for therapeutic interventions, the concept of facial emotion mimicry has broader applications, such as shared experiences during media consumption or in advertising contexts. These applications could provide valuable insights into the role of mimicry in social interactions and human-computer interfaces, representing an intriguing direction for future research.

## Methods

A feasibility experiment was designed to evaluate the proposed facial expression recognition as a therapeutic tool system in real-time. In this experiment, a facial point landmark algorithm estimated point landmark locations in 3D space from a USB web camera. This data was combined with geometric relations used as features for the recognition model. The aim was to evaluate facial expression recognition in real time, as well as collect data to improve on the model’s performance in post analysis. Participants were recruited and shown facial expressions performed by virtual avatars developed by one of the project partners (Imsimity GmbH, St. Georgen im Schwarzwald Germany). Participants were instructed at the start to mimic, similar to a mirror, the expression they saw the avatar express. The recognition model then ran the evaluation in the background and provided feedback to the user on how well they did and if they should repeat the task.

### Emotion recognition model

#### Feature extraction

To develop an efficient emotion recognition model resilient to variations in lighting and subject demographics, the geometrical approach to emotion classification was undertaken. Here, a point landmark detection algorithm was employed for estimating facial landmarks from which the different geometric features could be extracted. The selected algorithm was the Mediapipe^49^ framework for facial landmark detection, due to its robustness and capability to provide 3D landmarks. This algorithm adapted to facial orientation and distance relative to the camera, delivering 478 facial point landmarks encompassing the entire face and key features, such as the eyes, nose, mouth, and eyebrows. It also accounted for dynamic obstructions, such as a hand placed in front of the face^50^.

The choice to use Mediapipe^49^ was further motivated by its flexibility and lightweight design, making it well-suited for real-time emotion recognition tasks. Its robustness in facial landmark detection and adaptability to various facial orientations ensured high detection accuracy, while its open-source nature allowed seamless integration and customization within the experimental framework. These attributes made Mediapipe^49^ an ideal choice for tailoring the system to the specific objectives of this study.

**Fig. 5:**
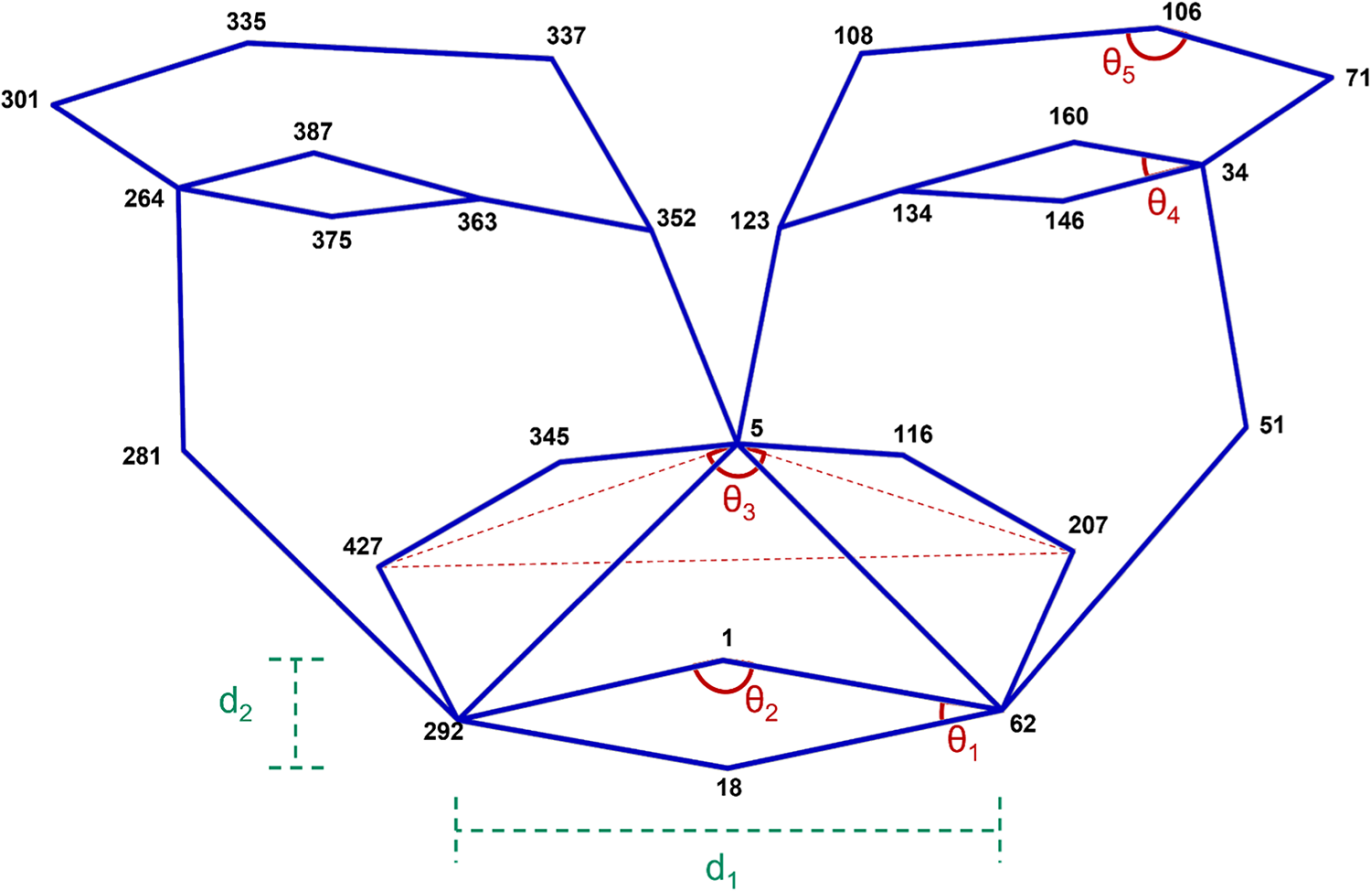
Reduced facial landmark points used. The point locations in point cloud are depicted in black bold numbers, feature angles are depicted in red, two out of the ten distance metrics in green. The blue lines represent the relation between the points that was undertaken.

Once the facial landmarks were extracted, a reduced number of points was selected based on^41^. Using the reduced points, geometric relations were set and the features of input for the recognition model generated. Figure 5 represents most of the points from the facial point landmark cloud that were used for the feature extraction. Table 2 shows the full range of point landmarks used for the study.

For the feature space, 15 features were used as input to the classification model, with a set of ten distance ratio metrics and five angles utilized. Table 2 lists the points used to calculate the Euclidean distance in 3D space as well as the points for calculating the feature angles for each individual frame.

**Table 2:** Features extracted to be used for the recognition model during the study. The term n.a. represents ‘not applicable’.

Feature name	Landmark point 1	Landmark point 2	Landmark point 3
d_1_	1	18	n.a
d_2_	14	15	n.a
d_3_	62	292	n.a
d_4_	79	309	n.a
d_5_	5	292	n.a
d_6_	62	5	n.a
d_7_	5	106	n.a
d_8_	335	5	n.a
d_9_	5	51	n.a
d_10_	281	5	n.a
θ_1_	1	62	18
θ_2_	292	1	62
θ_3_	427	5	207
θ_4_	160	34	146
θ_5_	108	106	71

The distance between the points was calculated using the Euclidean distance in Eq. (1). The angles were calculated using Eq. (2). $n$ represents the feature number for distance $d$, and $m$ represents the feature number for the angles $\theta$. $px$, $py$ and $pz$ represent the x, y, and z coordinate, respectively, of the particular point.

1 $${d}_{n}=\sqrt{{\left({px}_{1}-{px}_{2}\right)}^{2}-{\left({py}_{1}-{py}_{2}\right)}^{2}-{\left({pz}_{1}-{pz}_{2}\right)}^{2}}$$

2 $${\theta }_{m}= {\text{tan}}^{-1}\frac{\left({py}_{3}-{py}_{2}\right)}{\left({px}_{3}-{px}_{2}\right)}- {\text{tan}}^{-1}\frac{\left({py}_{1}-{py}_{2}\right)}{\left({px}_{1}-{px}_{2}\right)}$$

For the recognition model to be more robust to differences in people’s facial structure, the ratio of the distance features between a reference frame captured at the start of the experiment, which represents a neutral expression, and the current frame captured at the time of emotional mimicry study was computed and used as input for the recognition model alongside the angles from the current frame. This was calculated by Eq. (3) where ${R}_{n}$ is the distance ratio between the current and reference frame, ${d}_{nr}$ is the distance feature $n$ calculated at the reference frame, and ${d}_{nc}$ is the distance feature $n$ calculated at the current frame.

3 $${R}_{n}=\frac{{d}_{nr}}{{d}_{nc}}$$

#### Classification model

For the classification of the emotions, a simple pattern recognition model was implemented. The classification model included an input layer, one dense layer with output size 10, a sigmoid activation function and a classification layer i.e., a dense layer with output size same as number of classes and a SoftMax activation. The model was trained on 200 epochs with an adaptive moment estimation (adam) optimizer, a batch size of 128 and an initial learning rate of 0.01.

**Table 3:** Conversion of the six-class emotion and neutral expression representation to a two-class representation.

Original Class Name	New Class Name
Anger	Negative
Disgust	
Fear	
Sadness	
Happiness	Positive
Surprise	
Neutral	

The dataset was also split with a ratio of 90/10 for training and validation set respectively. To evaluate the performance, the validation sets the true positive accuracy was used and the confusion matrices visualized.

The six-class emotion representation along with the neutral expression was transformed into a two-class system of positive and negative classes based on the conditions depicted in Table 3.

### Dataset and data collection

The widely recognized facial expression recognition databases of OULU-CASIA^46^ and FACES^47^ were selected for the training and initial validation of the network models. The OULU-CASIA database was created with two image acquisition systems (infrared and visible light) under three different lighting conditions (strong, weak, and natural light). A subset from the database was chosen for the analysis. Specifically, the non-cropped RGB images taken under strong illumination. The data comprised a set of image sequences from 80 subjects portraying the six basic emotions: anger, disgust, fear, happiness, sadness, and surprise. The sequence begins with a weak representation and ends with a strong representation of the particular emotion. The strongest representation of each subject for each emotion was used for the analysis, while the first frame was used as the reference frame for the feature extraction for the model training. This subset had a final count of 480 emotion images and 480 neutral weak/neutral expression with an image resolution of 320 × 240 pixels.

The FACES database, was then combined with the data from the OULU-CASIA to encompass different subject demographics for an improved model robustness. The FACES database consists of facial portraits from subjects of various ages, with two images per emotional expression, covering the five basic emotions: anger, disgust, fear, happiness, and sadness as well as an additional neutral expression. The neutral expression from the FACES dataset for each subject was used as the reference frame to extract the features for the model training. Therefore, the selected database subset included a total of 1,710 emotion images and 342 neutral expressions, with a resolution of 2835 × 3543 pixels.

Finally, data were collected during a preliminary beta phase of an experimental study. Participants (8 in total, 7 male and 1 female) volunteered for the experimental trial of the proposed mimicry system developed for emotion training. The data collected was later used to test the robustness of the system and modify model approaches. The visual data from a camera was collected at a resolution of 480 × 640. The data size varied based on the number of attempts the subject performed to get a correct reading from the mimicry experiment. The details of the experimental setup can be seen in the “Experimental setup” section.

### Experimental setup

The conducted experimental trial involved having the participants sit in front of a monitor. In the first, ‘emotion assessment’, stage, participants attempted to identify the emotion that is portrayed by an avatar on the screen. In the second stage, to be referred to as emotion mimicry, the participants were asked to mimic the avatar’s facial expression, and were given feedback on their ability to recreate the expression. The system setup was separated into two sections, one for the participant and the other for the administrator.

**Fig. 6:**
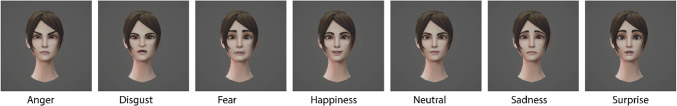
Different Avatar facial expressions displayed to the subject.

The avatars depicted the six basic emotions of anger, disgust, fear, happiness, sadness, and surprise as well as a neutral expression. Figure 6 represents the different avatars’ emotional expressions (anger, disgust, fear, happy, neutral, sad, and surprise) used during the study. The avatars were designed and developed by Imsimity GmbH (St. Georgen im Schwarzwald Germany). The design of the expressions was based on the facial action coding system (FACS)^45^, which is a widely adopted popular method for modeling and identifying facial expressions. The FACS provides a comprehensive and standardized framework for identifying and describing facial movements. By breaking down expressions into individual action units (AUs), which correspond to specific muscle movements, FACS allows for replication of human emotions. This level of detail enhanced the realism and accuracy of avatar facial expressions, making them more relatable and capable of conveying a wide range of emotions effectively.

#### System workflow emotion assessment

**Fig. 7:**
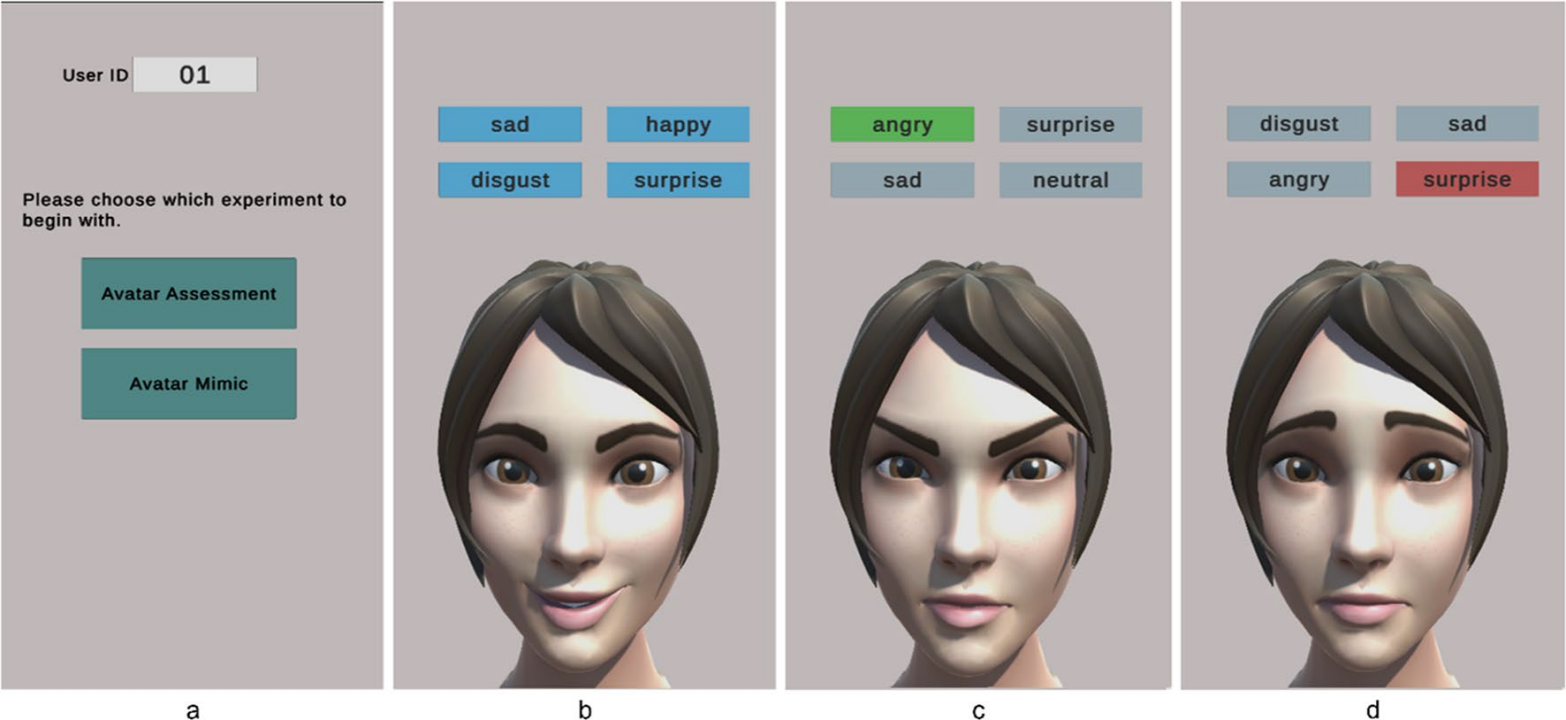
Participant view of the start interface (left **a**), the first screen after clicking on the assessment experiment (**b**), choosing the correct response (**c**), and in case of incorrect response (**d**).

In the participants’ section the user only sees the interface of the proposed system. The user is able to select from the stages of the experiment at any order they choose. In the assessment stage the user was prompted with four selectable options in which each option is set to an emotional category. An avatar then appears with a randomly selected pre-defined facial expression representing a particular emotion from the 7 pre-set expressions defined. The user can then select the emotion they most relate to the avatar’s expression. Among the four options given, only one was correct in which it would be highlighted green when the subject assumed it correct or red when the subject guesses wrong. This step was repeated for the different facial expressions defined. The order was randomized for each user. Figure 7 shows the participants view perspective at the launch of the system and during the assessment task.

#### System workflow emotion mimicry

**Fig. 8:**
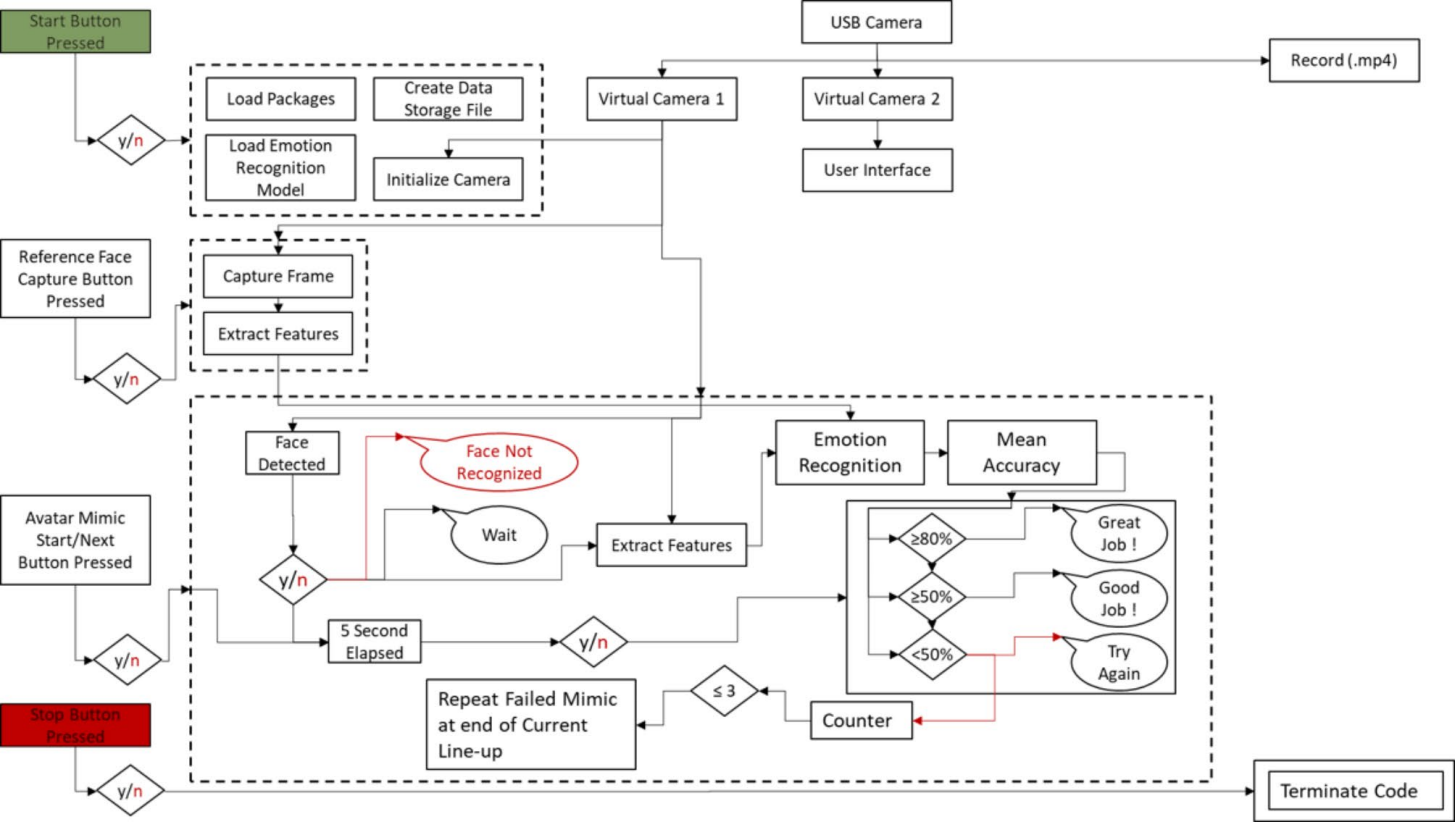
System workflow.

Figure 8 represents the system workflow for the emotional mimicry developed for the emotion recognition interface. The workflow outlines the sequence of steps from data acquisition to emotion recognition and feedback processing. A detailed description covers each step, including the conditions and actions taken at each phase, to provide a comprehensive understanding of the system’s operation.

**Fig. 9:**
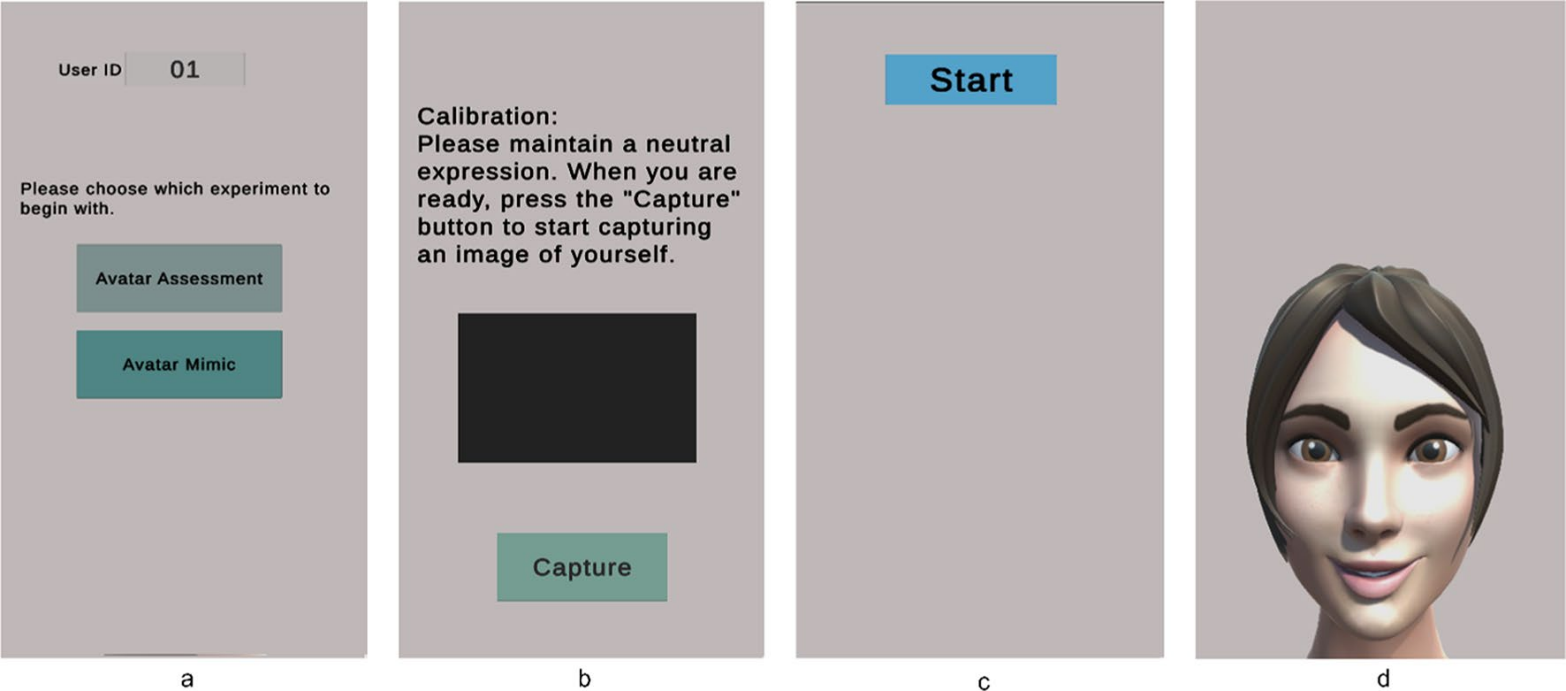
Participant view of start interface after completion of assessment experiment (**a**), screen for the capture of the reference frame with a live camera feedback depicted as black rectangle (**b**), transition phase where user feedback is requested to begin the emotion recognition phase (**c**), and the avatar facial expression depicted that the participant is asked to mimic (**d**).

**Fig. 10:**
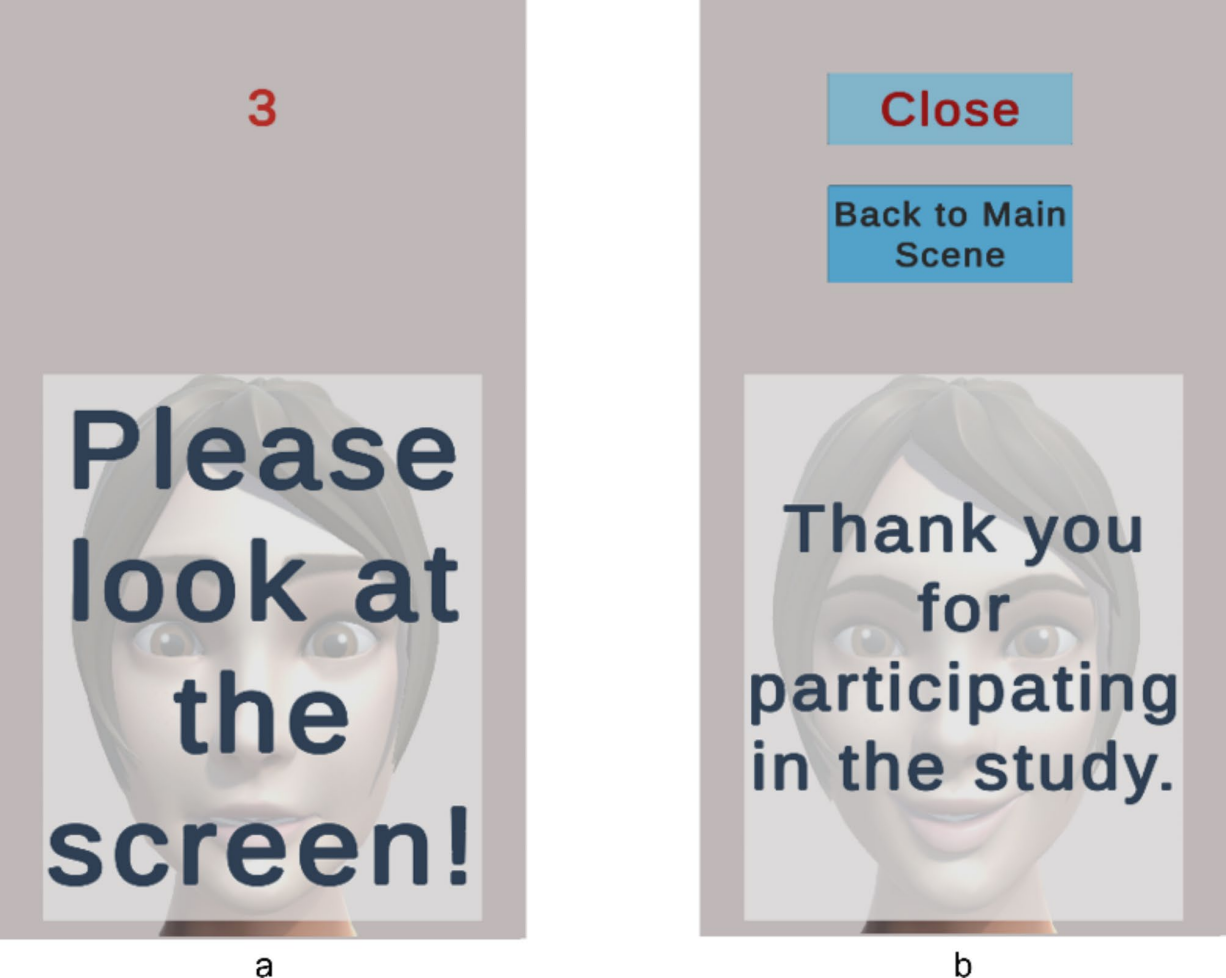
Participant view when the face is not recognized (left **a**), view at the end of the experiment (**b**).

**Fig. 11:**
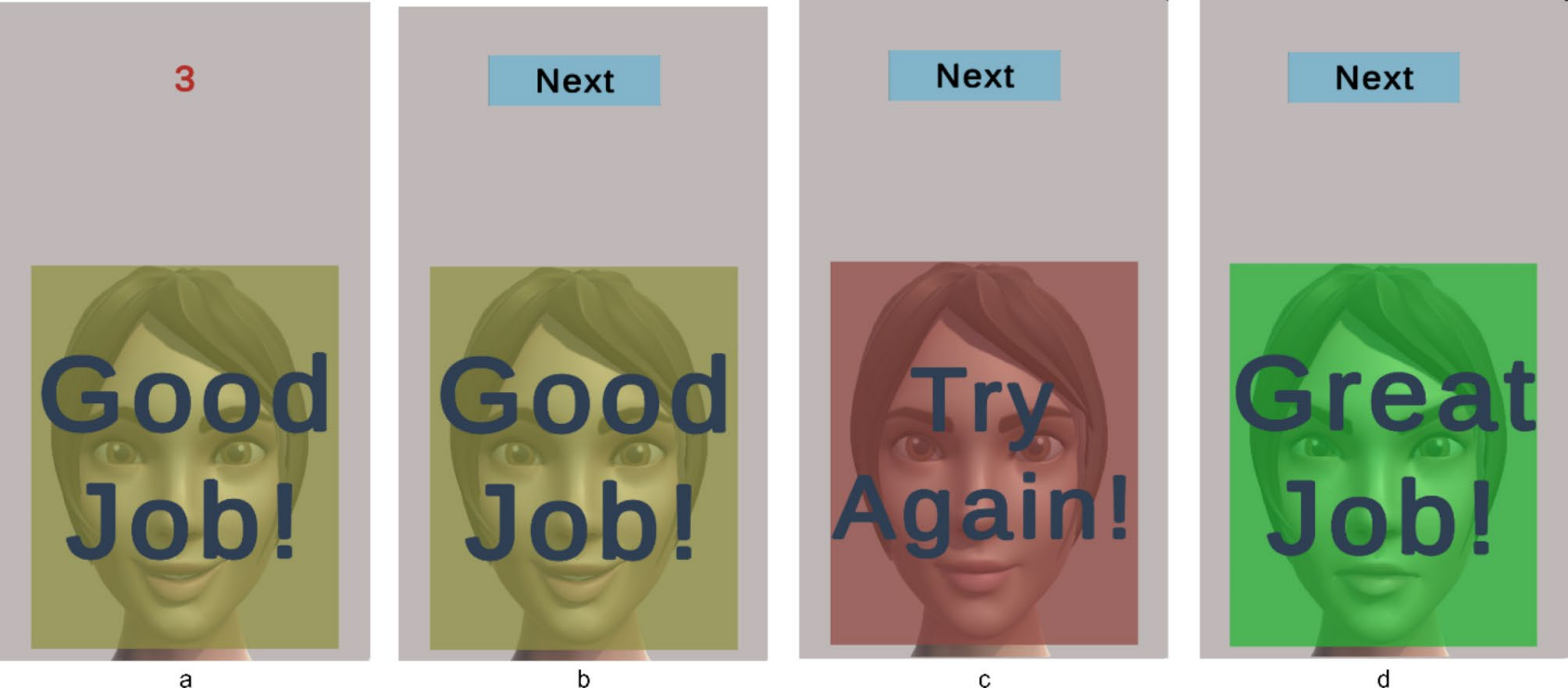
Participant view of the feedback from their mimic accuracy (between 80 and 50%) and countdown in red lettering to the next facial expression (**a**), screen after the cool down period is elapsed and the next button displayed (**b**), the feedback from their mimic accuracy (below 50%) (**c**), and feedback from their mimic accuracy (above 80%) (**d**).

- Start Button pressed:
  The workflow began when the user pressed the “Start Button” This action initiates the entire process, setting the system to start acquiring data from the user. Figure 9a represents the screen at the start of the interface after already having completed the assessment experiment.
- Initialization:
  Upon pressing the start button, the system performed initialization procedures. This involves loading the pre-selected trained recognition network model, creating a data recording file, loading the necessary packages for the software to run, ensuring all devices (camera) and applications (emotion recognition model, data recording) are ready for operation.
- Reference frame capture button pressed:
  After the initialization step was complete, the button to capture the reference frame (i.e., neutral expression) of the subject was visible to the user with a display showing the view from the camera. This display allowed the user to adjust his position as one does typically when standing in front of a mirror. This is visualized in Fig. 9b which represents the screen at the start of the reference capture process.
  After the button was pressed the system takes a snapshot of the image from the camera and extracts the relevant facial features required for the emotional recognition model to process. After this processing is complete the user was then migrated to the next scene of the interface where a “Start” button was displayed, that would take the user to the avatar facial expressions and begin the emotion recognition process. This is seen in Fig. 9c which represents the screen at the phase post reference frame capture and prior to emotion recognition start.
- Avatar mimicry start button pressed:
  The system then proceeds to display the avatar and a corresponding facial expression. The user is asked to mimic the expression for a period of 5 s. This can be observed in Fig. 9d where the avatar is shown with a specific facial expression. The system then internally proceeds to detect the user’s face using the visual information from the camera. This step is crucial as the subsequent emotion recognition process relies on accurately identifying and capturing the user’s facial features. If the face was detected successfully, the workflow advances to the next step. If not, the system generates a “Face Not Recognized” notification, prompting the user to adjust their position to ensure proper detection.
- Face recognition and data capture:
  Once the face was detected, the system captured the current frame. This frame was then used to extract the relevant features needed for the emotion recognition model. This process was performed on a continuous basis throughout the duration (i.e., 5 s) of the current emotional depiction by the avatar. An idle period of 3 s was set as a transition phase between the different facial expressions, as seen in Fig. 11a, where after receiving feedback a cool down period of 3 s shown as a countdown in red is displayed before the subject can move on to the next phase.
  If at any point the system fails to recognize the face again, it triggered a notification and attempts to re-establish proper detection and data capture. This is represented in Fig. 10a where the message is displayed to the participant with a timer countdown in red lettering at the top of the display.
- Emotion recognition model:
  The generated feature data were then processed by the emotion recognition model, which analyzed the facial expressions to classify the user’s emotional state. The model was pre-trained to identify specific emotions based on the geometric features of the face. The recognition model ran in near real-time, continuously updating its predictions as new data was captured.
- Data processing and validation
  The emotion recognition results are processed and recorded, and the system validated these predictions against the ground truth data that were the pre-defined avatar expression, to provide an average accuracy reading that would prompt for the appropriate feedback response. If the predictions are above a certain threshold accuracy, a feedback message was displayed to the user. The feedback of “Great Job!”, “Good Job!” and “Try Again!” is sent to the subject based on the mean accuracy being equal to or above 80%, between 50% inclusive and 80%, and less than 50% respectively. The workflow then proceeds to the next stage at the users’ pace, i.e., via a “Next” button press. If predictions are not above the threshold of 50%, the system would reinitiate the emotion recognition process, using the facial expression of the failed attempt, at the end of the current lineup of emotional expressions. The subject has at most 3 attempts to mimic the avatar expression if they fail to reach a minimum desired threshold. In Fig. 11b–d the different feedbacks are displayed.
- Result storage and display
  The experiment results were recorded and stored for future model enhancements as well as for post processing. This data included detailed logs of the recognized emotions, timestamps, and other relevant metadata.
- Stop button initialized
  The workflow concludes when the entire sequence of facial expressions was completed in which the “Stop” button is initialized internally. This action terminates the data acquisition and emotion recognition processes. This process was run in parallel to the user viewing the end of the study screen, as shown in Fig. 10b.
  After saving the final results, the system disconnected from the camera and software components used during the process. This step ensured no data were lost, and the system was ready for the next use.

#### Inter-program communication

**Fig. 12:**
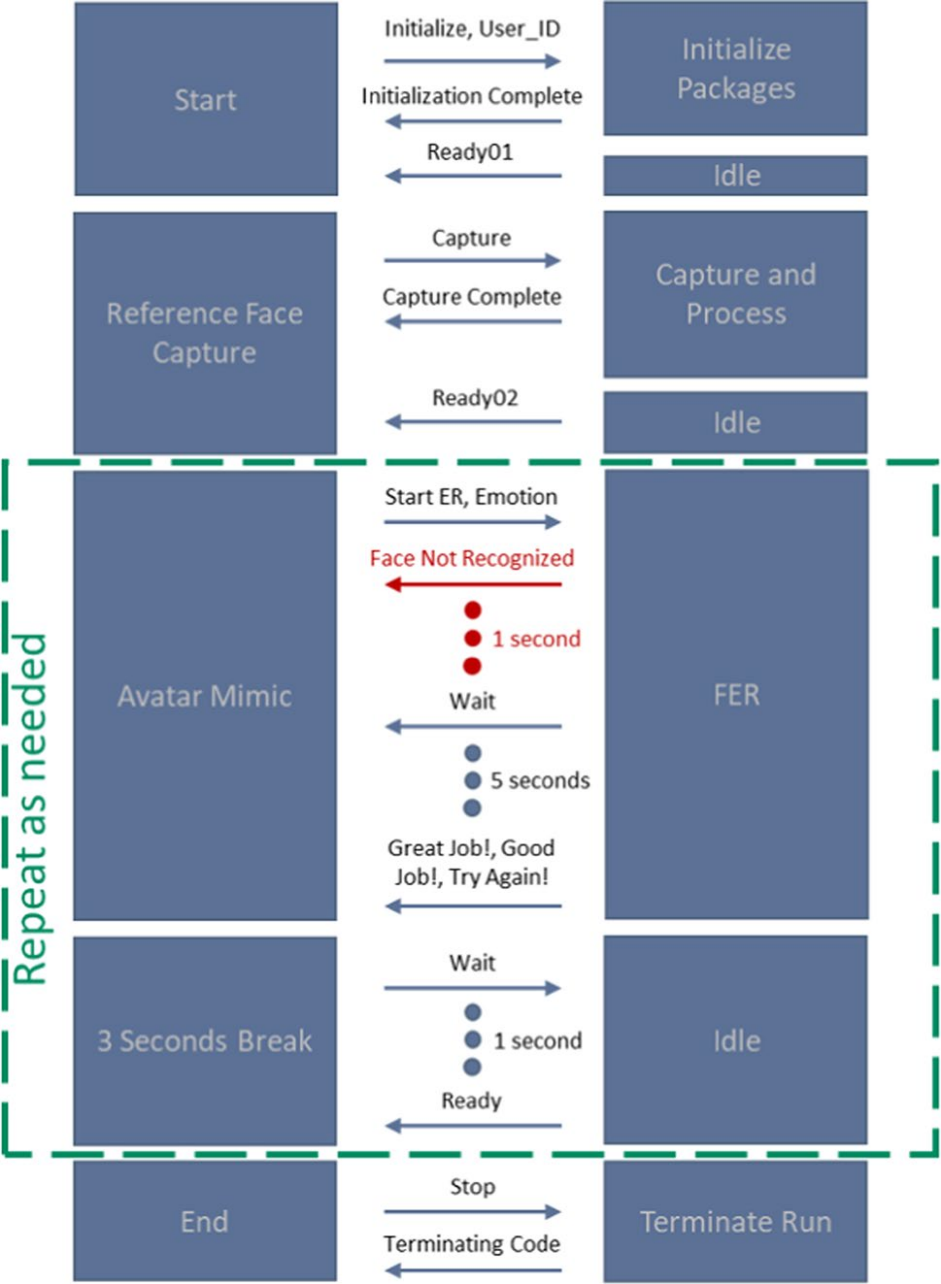
Communication commands sent and received between both platforms, C-sharp (c#) and Unity on the left, and python on the right.

The system was designed with two programming languages, python and c#. The user interface was developed using the Unity (Unity Technologies) program which runs in conjunction with c#, while the code and the recognition models were developed and analyzed using python. A suitable communication between the two platforms was required and therefore a communication protocol was established via 2 “.txt” files. In Fig. 12 the communication commands and phases of the system workflow are depicted.

During the mimicry experiment, when the user first selects the experiment by pressing the button the command of “Initialize” as well as the user ID set by the admin is sent to the recognition system to begin the subsequent steps necessary. After the initialization is complete, time for completion 30 milli-seconds (ms), the command “initialization complete” is sent followed by a “Ready01” command after 2 ms and sets the system in idle mode waiting for the user to do the next step.

When the user then presses the “Reference Frame Capture” button a command “Capture” was sent to the system to take a snapshot of the visual data and process the information to retrieve the relevant features from the reference frame and store it for use in the model recognition task. After the capture is completed and the features extracted and stored, duration of 1 s (s), a follow-up command of “Ready02” is sent to the interface within 2 ms and the system goes back into idle mode.

Once the “Start” button is pressed to begin the mimicry sequence, a command of “Start ER” along with the avatars pre-set emotion class was sent to the system. This emotion was used as the ground truth to assess the subject’s ability to mimic the expression. If the face was not recognized, a command is sent back to the interface to notify the subject and a delay of one second was set in order to provide ample time for the subject to adjust his position or head orientation. The emotion recognition would then run continuously for a period of 5 s. After which feedback consisting of “Great Job!”, “Good Job!”, and “Try Again!” was provided to the subject based on the mean accuracy of the predictions against the ground truth over the 5 s period.

After the time period has elapsed and the feedback is displayed to the user on the interface the system and interface perform a 3 s idle period in which the commands of “Wait” and “Ready” are exchanged after second. Then depending on the users pace the next emotion avatar was depicted and the process was repeated for the entire sequence of emotions, totaling 7 times in this case. If the subject failed to reach the threshold and has a “Try Again!” feedback, the failed emotion mimicry would be repeated again after the completion of the sequence. This repeat will happen up to a maximum of two times, therefore having each emotion repeated a maximum of 3 times in total.

Once the sequence was finished, the user was then notified on the interface and the command of “Stop” is sent to the system to shut down and terminate operations. The system then proceeds to send the command of “Terminating code”, after which it performs the necessary data recordings and closures of operations.

#### Hardware components

**Fig. 13:**
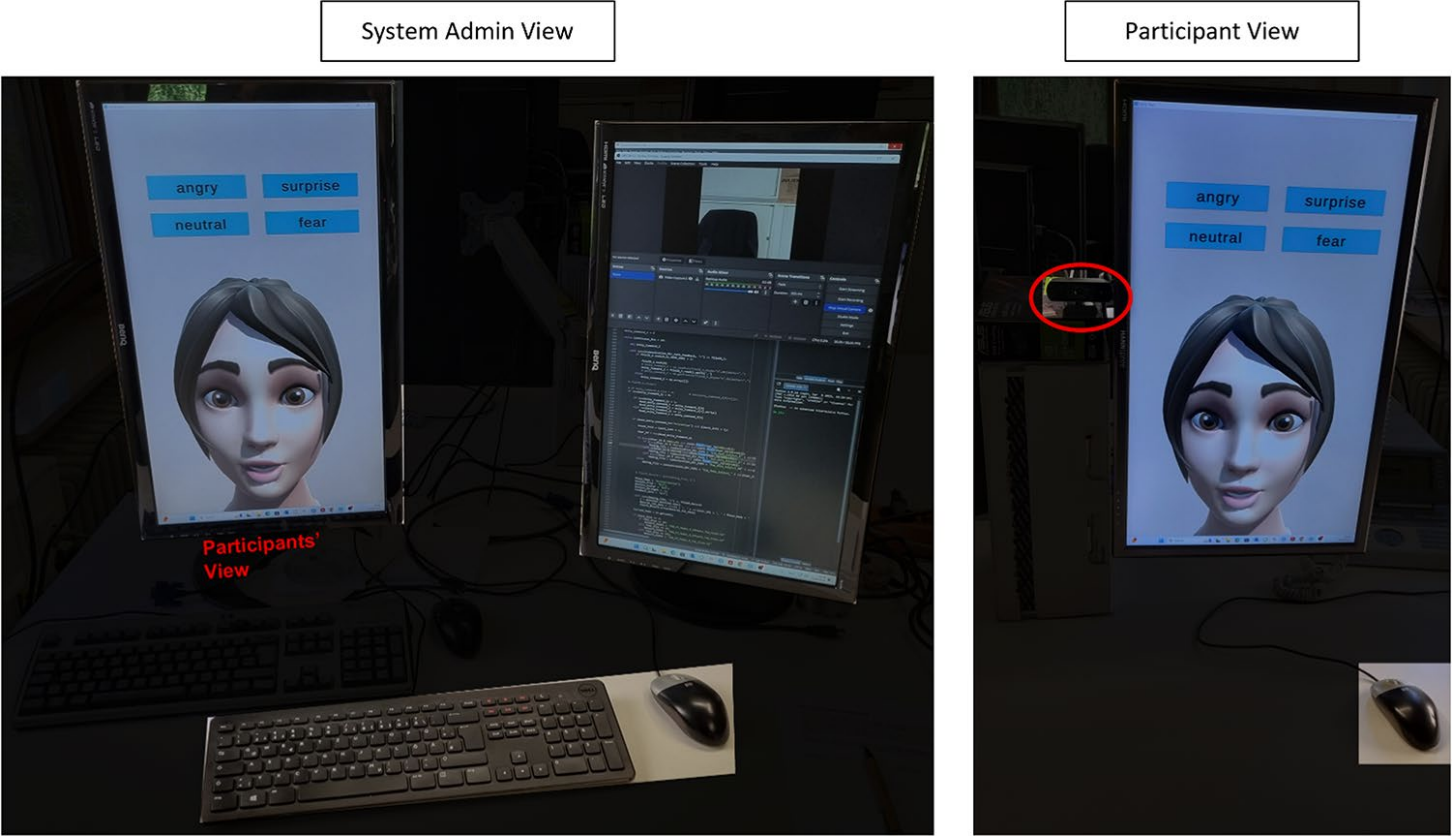
Participant view right and administrator view left of hardware components used for the experimental study. The camera is depicted by the red oval in the participant view.

The participant section included a monitor with a resolution of 1080 × 1920, a USB camera with a resolution of up to 1920 × 1080, and a computer-mouse for giving feedback to stage 1 of the experiment. The administrator section was composed of two monitors of resolution 1920 × 1080, and a computer-keyboard and mouse. Figure 13 displays the system setup of both participant and administrator view.

The experiment was conducted on a desktop with an AMD Ryzen 7 3700 × 8 core processor, 64.00 GB memory (RAM) and an NVIDIA graphics card RTX 3070 (NVIDIA Corporation, Santa Clara, CA, USA).

#### Software components

The software used in this experimental feasibility study included Unity (Unity Technology) for avatar display and interaction recording, Python for emotion recognition and data recording, and OBS Studio (Open Broadcaster Software) for virtual camera setup and video recording. The use of virtual cameras allowed for the reduction in the number of hardware cameras required.

## Data Availability

The datasets generated during and/or analyzed during the current study are not publicly available due to privacy, legal and ethical restrictions but are available from the corresponding author on reasonable request pending review.
